# A Rapid Realist Review of Effective Mental Health Interventions for Individuals with Chronic Physical Health Conditions during the COVID-19 Pandemic Using a Systems-Level Mental Health Promotion Framework

**DOI:** 10.3390/ijerph182312292

**Published:** 2021-11-23

**Authors:** Lorna Stabler, Maura MacPhee, Benjamin Collins, Simon Carroll, Karen Davison, Vidhi Thakkar, Esme Fuller-Thomson, Shen (Lamson) Lin, Brandon Hey

**Affiliations:** 1 School of Social Sciences, Cardiff University, Cardiff CF10 3NN, UK; 2 School of Nursing, University of British Columbia, Vancouver, BC V6T 2B5, Canada; maura.macphee@ubc.ca; 3 Rady Faculty of Health Sciences, University of Manitoba, 750 Bannatyne Ave, Winnipeg, MB R3E 0W2, Canada; Benjamin.Collins@umanitoba.ca; 4 Department of Sociology, Cornett Building, University of Victoria, Victoria, BC V8W 3P5, Canada; scarroll@uvic.ca; 5 Health Science Program, Kwantlen Polytechnic University, 12666 72 Ave, Surrey, BC V3W 2M8, Canada; karen.davison@kpu.ca (K.D.); vidhi.thakkar@kpu.ca (V.T.); 6 Factor-Inwentash Faculty of Social Work, University of Toronto, 246 Bloor St W, Toronto, ON M5S 1V4, Canada; esme.fuller.thomson@utoronto.ca (E.F.-T.); lamsonlin.lin@mail.utoronto.ca (S.L.); 7 Mental Health Commission of Canada, 350 Albert Street, Suite 1210, Ottawa, ON K1R 1A4, Canada; bhey@mentalhealthcommission.ca

**Keywords:** rapid realist review, mental health interventions, chronic physical health conditions, COVID-19, mental health promotion

## Abstract

The 2020 global outbreak of COVID-19 exposed and heightened threats to mental health across societies. Research has indicated that individuals with chronic physical health conditions are at high risk for suffering from severe COVID-19 illness and from the adverse consequences of public health responses to COVID-19, such as social isolation. This paper reports on the findings of a rapid realist review conducted alongside a scoping review to explore contextual factors and underlying mechanisms or drivers associated with effective mental health interventions within and across macro–meso–micro systems levels for individuals with chronic physical health conditions. This rapid realist review extracted 14 qualified studies across 11 countries and identified four key mechanisms from COVID-19 literature—trust, social connectedness, accountability, and resilience. These mechanisms are discussed in relation to contextual factors and outcomes reported in the COVID literature. Realist reviews include iterative searches to refine their program theories and context–mechanism–outcome explanations. A purposive search of pre-COVID realist reviews on the study topic was undertaken, looking for evidence of the robustness of these mechanisms. There were differences in some of the pre-COVID mechanisms due to contextual factors. Importantly, an additional mechanism—power-sharing—was highlighted in the pre-COVID literature, but absent in the COVID literature. Pre-existing realist reviews were used to identify potential substantive theories and models associated with key mechanisms. Based on the overall findings, implications are provided for mental health promotion policy, practice, and research.

## 1. Introduction

The 2020 global outbreak of COVID-19 has highlighted the need to promote and support the mental health of individuals, families, and communities. The pandemic has introduced a mental health crisis due to pandemic threats, such as risk of exposure, physical and social isolation, financial insecurity, and physical and emotional fatigue [1,2,3].

Throughout the world, public health responses to COVID-19 were implemented to reduce viral transmission, leading to major disruptions in activities of daily living and increasing fear, anxiety, and other negative emotions [4]. Individuals with chronic physical health conditions have risks from COVID-19 that are disproportionately higher than the general population [5]. Greater risk for developing severe physical complications from COVID-19 is associated with decreased perceptions of quality of life and higher levels of depression, anxiety, and emotional distress [6]. One review across 93 countries highlighted how underlying chronic diseases are key population risk factors for COVID-19 mortality [7]. Another review paper [8] identified the following major mental health risk factors for individuals during COVID-19: (a) medical comorbidities, such as cardiovascular and pulmonary diseases, diabetes, obesity, which are risk factors for severe viral infection; (b) increased age; (c) cognitive and behavioural disorders that adversely affect compliance with rules; (d) stigmatisation and racism; and (e) lack of access to the social determinants of health. Individuals with chronic physical health conditions, therefore, are at high risk for poor mental health due to the potential for severe illness from the COVID-19 virus and from the adverse consequences of public health responses to COVID-19.

### 1.1. Background

A recent scoping review conducted by our team [9] focused on interventions to prevent and manage mental health challenges among those with a physical condition that presents risk of severe COVID-19 infection. Our scoping review revealed that anxiety and depression were the most commonly reported mental health conditions for individuals with chronic physical health conditions during COVID-19. Increased levels of adverse mental health conditions were associated with barriers to resources (e.g., health care team support); lack of control (e.g., ability to adhere to treatment regimen); or concern with contracting COVID-19 [9].

Effective mental health promotion depends on linkages between different systems levels: macro-level policymakers and governments, meso-level community services and primary care, and micro-level individuals with patients and family caregivers. This rapid realist review (RRR) is a further exploration of the contextual factors and underlying mechanisms or drivers associated with effective mental health interventions within and across macro–meso–micro systems levels for individuals with chronic physical health conditions who are susceptible to contracting COVID-19.

Under-funding and lack of strategic planning for mental health in all policies indicate that mental health is less of a policy priority than physical health [10,11,12]. The rapid and consuming nature of the COVID-19 pandemic and the mental health burden this has imposed, coupled with the chronically undervalued importance of mental health, situate this RRR paper as especially timely and important.

### 1.2. Study Aim

To contribute to understanding and mitigating the mental health impacts exacerbated by the COVID-19 pandemic, we conducted an RRR of documents from the scoping review. We employed a realist approach to undertake this review [13,14]. Realist approaches focus on which interventions work for whom and under what circumstances. Certain contextual factors (Cs) influence if/how key actors choose to use the intervention as designers expect and need them to do. The realist approach focuses on understanding the underlying mechanisms (M) related to actors’ choices and actions that ‘cause’ outcomes to happen (be they positive or negative in terms of intervention success). Realist approaches represent interactions between contexts (Cs), mechanisms (Ms), and outcomes (Os), known as CMO configurations. In this respect, realist approaches often present unique and novel perspectives for intervention outcomes that provide an understanding of why and how the interventions do or do not work [13]. Realist approaches are therefore well-suited to studies of complex systems such as the topic of this review.

**Figure 1:**
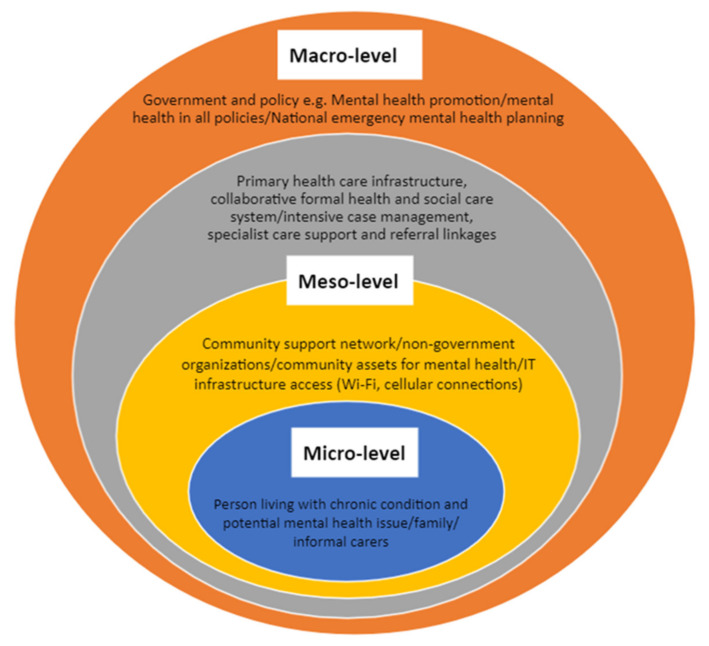
Mental health promotion framework or programme theory for this rapid realist review.

We used a systems-level model (i.e., macro–meso–micro) as a framework for interpreting our findings (Figure 1). A critical first step in realist approaches is the development of a programme theory that describes how a programme is considered to work [15]. The programme theory is comprised of CMOs that are tested and refined with evidence gathered from a review of relevant documents. We used the framework in Figure 1 as our programme theory, to better illustrate evidence-based contextual factors and mechanisms at every level and across levels.

The realist question addressed in this paper is: What mechanisms or drivers of human decisions and behaviours were associated with effective uptake and delivery of mental health interventions for individuals with chronic physical health conditions who are at risk of contracting COVID-19 and having severe illness at macro–meso–micro systems levels?

## 2. Materials and Methods

### 2.1. Search, Selection, and Appraisal Processes

We used RAMESES realist review publication standards to guide the phases of our RRR [15]. These standards have specific criteria for document selection and to determine relevance and rigor. The RRR was undertaken alongside a scoping review [9] that yielded key documents that we used to develop CMOs for each systems level within our initial programme theory (See Figure 1). The published scoping review includes inclusion and exclusion criteria and quality assessment criteria [9].

Realist approaches to document screening and selection differ from other review approaches [13,14]. Inclusion criteria focus on whether or not a document contains information about: (a) mental health interventions for the target population; (b) contextual factors associated with the interventions; (c) underlying mechanisms or human decisions/reasons influencing their use of interventions; and (d) proximal and/or distal outcomes associated with the interventions. In this way, reviewers focus on identifying papers that add conceptual richness [16]. All study types and grey literature are typically included (e.g., qualitative, quantitative, mixed methods, commentaries, editorials).

**Figure 2:**
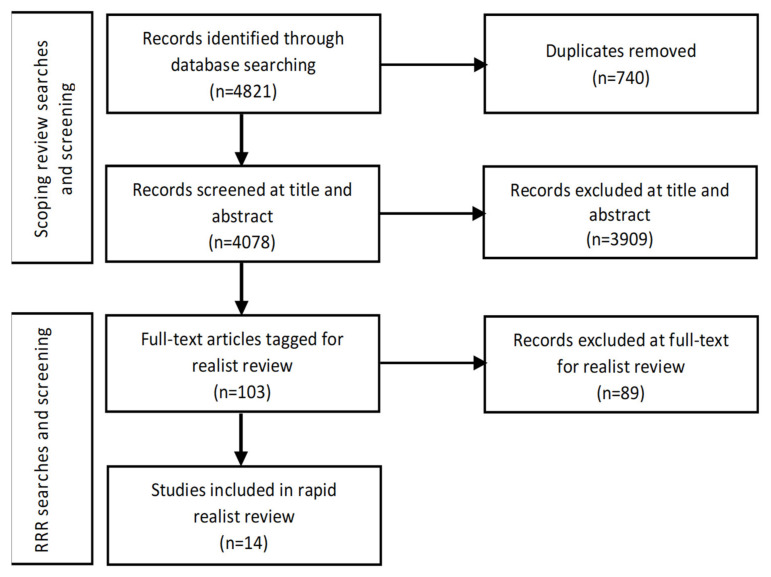
PRISMA diagram showing stages of the scoping and RRR search, screening, and selection process.

For selection, two reviewers (L.S. and M.M.) read all the scoping review abstracts to identify documents to include in the RRR. Papers that either reviewer identified as having data pertaining to CMOs for programme theory development were read at full text by both reviewers. Inclusion was decided based on whether papers included enough data for programme theory development [15]. Fourteen scoping review papers were included in the RRR. Figure 2 is the PRISMA diagram for the scoping and RRR document search, screening and selection process.

### 2.2. Data Extraction and Analysis

A data extraction template was developed by the team to more efficiently code for CMOs to populate our programme theory. We also used the literature to identify any substantive theories or models associated with key mechanisms. Because realist methods are used to develop and refine programme theories with their testable CMOs, an important component of realist methods is building on existing theories, typically mid-range theories from psychology and sociology. Existing theories help explain the functioning of underlying mechanisms—the drivers of actors’ choices and actions [13,15]. Data analysis was conducted by two independent researchers using NVivo software to code for CMOs and substantive theories associated with key mechanisms.

### 2.3. Patient and Public Involvement/Stakeholder Engagement

For the scoping review and the RRR, stakeholders representing each of the three systems levels of the framework were included throughout the project lifecycle to add rigor to the review process. Stakeholders helped to frame review questions, they provided useful literature from non-academic sources, and they attended fortnightly meetings to develop policy recommendations from the scoping review [9]. Stakeholders also contributed to validation of the programme theory and CMOs for the RRR.

## 3. Results

### 3.1. Study Characterisics

Overall, 14 studies were included from the scoping review papers to develop the programme theory and CMOs for the RRR. Table 1 includes the authors, year, country of origin, and topic of each document.

### 3.2. Programme Theory Development

Table 2 shows the CMOs constructed for macro–meso–micro levels. We identified four key mechanisms: trust, social connectedness, accountability, and resilience. These mechanisms operated at each systems level except for accountability, which was only present at the macro-level. The following sub-sections provide supporting literature evidence for each of the four mechanisms and their respective contextual factors and outcomes at each systems level. Our examples are taken from 10 documents that best illustrate CMO relationships [13,14].

#### 3.2.1. Trust

At the macro-level, trust as a key mechanism was predominantly associated with contextual factors that highlighted a government’s capacity to provide timely access to: (a) valid information (e.g., what to do, where to go for help during the pandemic); and (b) mental health support services [17]. During the pandemic, trust in the government’s capacity to meet public health needs during COVID-19 was undermined by delayed communications or miscommunications. In China, up-to-date, specific and accurate public health information was associated with lower levels of stress, anxiety, and depression as measured with public surveys [18,19]. In addition, public health messages provided concrete protective measures to avoid COVID-19 infection [20]. In Canada, an Angus Reid public poll showed that 33% of the polled sample was not confident that the public health system was prepared to handle the pandemic—at a time when only four cases of COVID-19 had been documented. Lack of trust in the government fueled fear of infection with consequent increases in anxiety, avoidance behaviours, and stigmatisation: these reactive fears and behaviours were seen globally in the absence of public trust in government leadership [17].

“The COVID-19 pandemic has made health services and policy makers rethink the way we deliver services and organise resources in the best possible ways.”

[17] (p. 23)

The National Health Commission of China quickly instituted psychological crisis prevention public health measures at the onset of the COVID-19 outbreak in Wuhan. At each level of government, funding was provided for online platforms to manage mental health consultations and prescriptions; mental health outreach teams in communities; and 24/7 mental health hotlines. “Telemental services” were particularly popular in more remote areas of China. Through government actions that acknowledged the psychosocial effects of the pandemic, public trust in the government was heightened [17].

Trust was also important between patients and primary care providers at the meso-level. One in five patients consult their primary care providers for emotional-social problems versus medical ones [20]. During the pandemic, primary care providers were ideally placed, through established trust relationships with patients, to screen at-risk individuals for signs of mental health exacerbation using standardised tools (e.g., anxiety [GAD-7], depression [PHQ-9]); to ensure continuity of care; and to convey important information about viral transmission, protection from infection and vaccination options [20,21]. Trust between patients and providers also enabled effective transitions between in-person consultations and virtual ones.

“Primary care doctors providing patient-centred, longitudinal care are in a unique position to provide psychological support and treatment during the current pandemic, since continuity of care is associated with lower mortality rates and better patient outcomes.”

[20] (p. 2)

At the micro-level, individual acceptance of alternative forms of care delivery depended on trusting relationships with their workers or teams. In Italy, for example [21] individuals with immune deficiencies were shifted from hospital-clinic to home support therapy. “Remote” visits were used to adapt the home care regimen as needed. Surveys with patients found that trust in their providers to individualise their care (even remotely) resulted in no changes (pre/during COVID-19) with respect to quality of life and levels of anxiety and depression. Individualised attention through trusted providers or workers, therefore, may be necessary at an individual level to assuage negative emotions associated with changes in treatment regimens or the introduction of new services.

#### 3.2.2. Social Connectedness

During the pandemic, people’s usual psychological needs for social connection were severely disrupted. At the macro-level, governments pledged extra funding to decrease adverse effects of social isolation on vulnerable populations [20]. The UK, for example, pledged extra funding to train and integrate additional “social prescribers” within the National Health Service (NHS) primary care provider networks (meso-level). The NHS social prescribing program began in 2018 with significant funding increases at the start of the pandemic to assist at-risk patients with connecting to community resources (e.g., counselors, peer supports, online exercise, and arts classes) [20]. Within the NHS, social prescribers are trained staff who work with primary care providers to promote social connectedness and mental and physical well-being. Previous research has shown that social prescribing is a cost-effective approach to “prevent a range of physical and mental health conditions” [20] (p. 3).

At the micro-level, social isolation during the pandemic resulted in mental health deterioration [22]. Those living alone had greater negative psychological effects from loneliness and isolation [23,29]. Older adults with pre-existing mental illness or cognitive impairment were susceptible to increased anxiety and behavioural problems, and one case study of an Alzheimer’s patient found that video-calling with family members decreased behavioural problems [24]. A phone survey study from the Ukraine found that during an extended COVID-19 lockdown, regular social support was necessary to ensure older individuals with substance use and chronic physical health conditions (e.g., HIV) continued to adhere to their medical treatment regimen. Pharmacological treatment interruptions were more common when social supports were lacking [25].

#### 3.2.3. Accountability

At the macro-level of policy and government, accountability was a key mechanism associated with timely, accurate government communications with the public. The term “infodemic” was coined during the SARS epidemic to refer to rapid spread of misinformation and forged news, primarily through social media platforms [18]. Infodemics can have serious negative public health consequences, such as vaccination hesitancy or refusal. Misinformation can also increase negative emotions, such as anxiety and fear [18]. To mitigate these risks during COVID-19, accountable public health officers provided factual and timely information through social and conventional media so that the public had a realistic appreciation of how to keep themselves safe from viral transmission [18]. None of the papers in this RRR discussed accountability at the meso- or micro-levels—something which we address in the Discussion.

#### 3.2.4. Resilience

Resilience was the final key mechanism discussed in the review literature with respect to all three levels. Resilience was defined in different ways, depending on the systems level. We will return to these differing definitions in the Discussion. At the macro and meso levels, resilience signified the capacity to be flexible and adaptable with limited time for preparation and planning during COVID-19. At the macro-level, resilient governments quickly lowered regulatory and communications barriers to enable physical health and social care service access for at-risk patients. For example, England and Wales made changes to the Care Act 2014 so that local authorities could prioritise and tailor services to specific vulnerable populations [17]. In the US, government agencies suspended Health Insurance Portability and Accountability Act (HIPAA) compliance and allowed healthcare providers to use popular applications, such as FaceTime and Facebook Messenger for video chats with clients [24]. Data protection and reimbursement laws were also amended for the pandemic [26]. Similarly, at the primary care meso-level, providers rapidly implemented virtual patient management, including mental health screening and mental health promotion referrals for at-risk individuals [26]. However, as will be discussed later, this was not necessarily done in partnership or consultation with those at different levels of the socio-ecological framework.

At the individual micro-level, resilience referred to coping strategies and capacity to reduce stressors that exacerbate mental and physical health conditions [22]. Effective coping strategies for individuals with chronic physical health conditions during COVID-19 were acceptance and self-distraction. Acceptance and self-distraction are considered positive, active coping styles. Interestingly, denial, a passive coping style, was also common among individuals with chronic physical health conditions who were surveyed during the pandemic. The researchers surmised that denial served as an effective short-term coping strategy to reduce stress [27]. Positive, adaptive coping strategies can be enhanced through psychoeducation and brief therapies that can be done virtually, such as cognitive behaviour therapy, exposure-based therapy, stress inoculation and relaxation training [18]. It is important to note that some sporting facilities—such as gyms and pools—that provide active self-distraction strategies, were shut down during COVID-19, necessitating re-direction of normal coping strategies. Social prescribing (described above) was one way to assist individuals with finding healthy self-distraction outlets [20].

### 3.3. Purposive Literature Search of Pre-COVID Realist Reviews

Based on stakeholder input, we were curious about whether or not the mechanisms we found in the COVID-19 literature were relevant to mental health promotion activities at the different systems levels prior to the pandemic. Because mechanisms represent triggers or ‘influencers’ of human decisions and actions to use specific programmes or interventions as intended, we did a purposive search of realist reviews on mental health interventions at the macro-meso-and micro levels pre-COVID. Realist methods employ an iterative process of searching literature to increase the rigor of preliminary findings [13,14]. With assistance from our research team and key stakeholders, we located 11 realist review papers (Table 3) that met RRR inclusion criteria. Two researchers independently read through the reviews and coded them for CMOs and substantive theories or models associated with the mechanisms that were relevant to those identified in the COVID literature. Realist reviews were the focus due to the rapid nature of the review—these papers clearly identified CMOs and theories related to their proposed mechanisms.

We identified five key mechanisms in the pre-COVID realist reviews: trust, social connectedness, accountability, resilience, and power. The following sub-sections provide a brief comparison of mechanism and contextual factors differences we found pre-COVID and during COVID, including the addition of a fifth mechanism, power-sharing. We also highlight mid-range theories associated with each mechanism within the pre-COVID realist review literature.

The following sub-sections reflect on our findings from the synthesis of the COVID-19 scoping review papers and our purposive search of pre-COVID realist reviews on our specific topic of mental health interventions. We believe this comparison illuminates how different contextual factors influenced some of the same key mechanisms we found during the pandemic (trust, accountability, social connectedness, and resilience) while one mechanism, power-sharing, was present in pre-COVID literature but not during COVID. Our Discussion provides our interpretation of these findings.

#### 3.3.1. Trust Pre-COVID-19 and during COVID-19

During COVID-19, public trust in the government (macro) and in primary care providers (meso) was vital for accurate messaging of public health pandemic management information (e.g., viral transmission, vaccinations) [17,18,19,20]. At the micro-level, established, trusted relationships between individuals/families and practitioners/support workers were necessary to ensure tailored adaptations to treatment plans [20,21]. Pre-COVID-19, the trust mechanism was described in relation to forming two-way social connections for sharing information and resources and making collaborative decisions [34,36] and at the micro-level for developing trusting relationships to facilitate engagement in interventions [35]. Contextual factors, therefore, differed. During COVID-19, contextual factors were related to acceptance of messages, information, and treatment plans, as where the focus on trust pre-COVID-19 was relational and dynamic in nature—giving and receiving. Diffusion theory [41] highlights the importance of participatory engagement, information sharing, and ‘diffusion’ throughout communities and individuals.

#### 3.3.2. Social Connectedness Pre-COVID-19 and during COVID-19

During COVID-19, social connectedness was manifested as transitions from in-person to virtual forms of communicating with people, especially at-risk individuals, such as people living alone and seniors with dementia or mild cognitive impairments [21,22,23]. Ensuring social support connections, especially for vulnerable individuals, was a priority of governments and community services and primary care providers [17]. Pre-COVID-19, social connectedness was contextualised as relationship-building among and between stakeholders at macro and meso levels [32,36,37] and as enabling individuals to connect with and self-manage their own mental and physical health needs at the micro-level [32,39].

Potential theories in the pre-COVID literature were cultural adaptation theory [42], trauma-informed models of care delivery [43], empowerment theory [44], and social network theory [45]. To build sustainable social connections with diverse populations and communities, there needs to be sensitivity to cultural contexts [42], barriers to access [38], and past traumatic events [39] when co-designing mental health interventions at each systems level (i.e., policy, community, individual). Empowerment theory explains how individuals’ strengths and capabilities can be harnessed for advocacy and social change (e.g., proactive community behaviours) and for greater personal control at the micro level [44].

#### 3.3.3. Accountability Pre-COVID-19 and during COVID-19

During COVID-19, accountability referred to government commitment to deliver accurate and timely information to curb misinformation (i.e., infodemic) and negative emotions, even panic, among the public. The focus in the COVID-19 literature was reactive—controlling the spread of misinformation and getting critical resources out as quickly as possible to manage viral transmission and treatment [18]. The roles of communities, providers and individuals were not described: instead, it was ‘a given’ that the government would be directing information and resource flow down—with no discussion of stakeholder input upwards.

Prior to the COVID-19 pandemic, accountability was related to commitment from stakeholders at different levels—government (macro), community services, and primary care providers (meso)—to engage in participatory models of designing, implementing and evaluating mental health interventions [30,33,36]. Relational aspects of working together within and across systems levels were highlighted in the realist review literature. At the individual micro level, individuals’ capacity to manage their own mental health needs and to take advantage of available services required accountability to themselves and others. The authors of one review paper described accountability as a pre-cursor to self-empowerment and eventual self-management [36]. According to another review [39].

“Programs and program strategies that support autonomy and self-directions in treatment and use of services will likely lead to longer-term positive health changes compared to programs that are fixed…”

(p. 983)

Community engagement models at the macro and meso levels [37] and empowerment theory at all three levels [44] were most prevalent in this body of realist review literature.

#### 3.3.4. Resilience Pre-COVID-19 and during COVID-19

During COVID-19, the resilience mechanism focused on the capacity for government authorities and healthcare organisations and providers to reorganise structures and processes based on public health needs [17,18,23,25]. At the individual level, resilience referred to individuals’ capacity to cope effectively with ongoing challenges and stressors [21]. Although the COVID literature seemed to equate resilience with quickness and adaptability, one earlier paper based on the SARS and influenza experiences in Canada [46] looked at resilience in relational terms. According to these authors, the most resilient organisations generated relational reserves prior to stressful events through collaborative sharing and engagement with key stakeholders [46].

Resilience in pre-COVID realist reviews was often associated with power-sharing and co-production, discussed in the following section. One pre-COVID realist review discussed resilience primarily at the neighborhood or meso-level, where community engagement theory was used to describe how community assets are necessary to add resilience or to buffer against stressors, such as lack of access to social determinants of health [40].

#### 3.3.5. Power-Sharing

Pre-COVID realist reviews identified power-sharing as a key mechanism at each systems level [30,36,37]. This is discussed in relation to empowerment theory [44] and community engagement theory [40,47]. For example, a civic engagement realist review [36] described empowerment as shared decision-making between community providers and service users.

“Civic engagement has the potential to transform mental health systems…It can also lead to improved information about, and access to, mental health care as well as enhancing relationships between patients and clinicians.”

(p. 2)

At the micro-level, a contextual factor associated with empowerment of individuals was access to integrated health and social care services; to improve clients’ access to the social determinants of health [30,37].

In the COVID literature for this RRR, the power-sharing mechanism was absent at all three levels—perhaps due to the switch-over to crisis management where decisions and communications became more top-down [48]. The absence of power-sharing and relationship-building in the COVID literature is notable, since these mechanisms are often theorised as being pre-requisites for resilience.

## 4. Discussion and Implications

Our RRR yielded four key mechanisms associated with effective mental health interventions during the COVID-19 pandemic: trust, social connectedness, resilience, and accountability. Our programme theory (Figure 1) and CMOs (Table 2) suggest that these four mechanisms enable each other within and across each systems level (i.e., macro–meso–micro), depending on the associated contextual factors. We conducted a purposive search of pre-COVID realist reviews on our study topic, looking for evidence of the robustness of these mechanisms. Trust and social connectedness were prevalent pre-COVID and during COVID with respect to mental health promotion at all systems levels. During COVID, accountability functioned at the macro-level with respect to top-down policy for pandemic management and communications, while pre-COVID, accountability was associated with commitment to mental health promotion at each systems level. In the COVID literature, resilience referred to responsiveness and adaptation of the government, providers and individuals. In the pre-COVID literature, resilience was associated with power-sharing and collaborative decision-making with respect to mental health interventions for communities and individuals. Our stakeholders pointed out that resilience can be a contested term when it is used to shift responsibility for health to individuals. Power-sharing, therefore, may be a necessary precursor for resilience, to ensure adaptation and support for mental health promotion that engages with intended users at every systems level.

Not surprisingly, the most common substantive theory associated with the power-sharing mechanism was empowerment theory [44]. The lack of power-sharing in the COVID literature suggests displacement of collaborative, shared decision-making during the pandemic. This is particularly problematic given long-standing issues related to trauma-informed or culturally safe care—when the voices of those with lived experiences must be attended to [30,39]. The CMOs and the conceptual models and theories from previous mental health intervention realist reviews support the importance of investing in empowering, participatory, collaborative strategies with key stakeholders to create necessary relational reserves. Resilient systems during pandemics, therefore, may depend on pre-crisis, sustainable relational investments.

Although our RRR was focused on mental health interventions for individuals with chronic physical conditions at risk of severe illness from COVID-19, we believe the underlying mechanisms and the substantive theories associated with them are universal ingredients for mental health promotion: linking policy to community to service users within and across macro, meso, and micro levels of health systems. Our purposeful comparison of pre-COVID realist reviews suggests the enduring importance of the mechanisms identified as underpinning effective mental health interventions during COVID.

### 4.1. Implications

Table 4 includes policy and practice recommendations for practitioners and policy makers with respect to the design and delivery of mental health interventions. We have included the four key mechanisms from the RRR and a fifth mechanism, power-sharing, from the pre-COVID literature. Our stakeholders believe that all five mechanisms may be necessary for delivering mental health interventions for those with chronic illness. All five mechanisms, therefore, need to be tested in real world settings to understand further how they interact with each other within and across systems levels to influence effective mental health promotion for populations with physical health conditions and high risk from COVID-19.

Future research is needed to test the findings of the review; its emerging programme theory and CMOs in different settings (e.g., different healthcare sectors, different user populations, different countries of origin). Reviews are based on secondary data and evaluations collect primary data to test the CMO ‘explanations.’ In complex settings, realist evaluation is an appropriate approach for exploring “What works for whom in what circumstances and in what respects, and how?” [14]. We believe the initial evidence from this RRR warrants further testing with realist evaluation. More research is needed to understand how these mechanisms serve as linkages across the three systems levels, which contexts moderate the mechanisms, and which outcomes occur. Initial evidence from the RRR indicates that these mechanisms (Table 2) are interrelated and present at each systems level. Our next steps, therefore, will be to test the programme theory and the CMOs from each systems level, ideally using mixed methods with a specific population, such as one municipality.

### 4.2. Strengths and Limitations

The rapid realist method is not aimed at conducting exhaustive searches, although realist approaches require iterative searches of different bodies of literature to refine programme theory and its CMOs. Given our RRR timeline, our work exposed many contextual factors, mechanisms, and supporting theories and models that will require further investigation. Our Discussion Section includes some of the additional literature we began to investigate more deeply after completion of our RRR with scoping review literature [9]. Because realist methods are theory-driven, we think we have a good starting point for ongoing investigation.

An incredible challenge during this RRR was the unprecedented rate of COVID literature generation. This means there may be studies that were not included in this RRR. However, the current study was extensive, and conducted on many literature search engines to identify a large range of available sources via the scoping review. Furthermore, on-going discussions with research team and stakeholders helped refine our RRR findings and create potential new avenues for literature searches to inform our programme theory and CMO development. For example, one stakeholder–researcher team meeting resulted in our search of previously published realist reviews to make best use of pre-COVID programme theories, CMOs, and proposed supporting theories and models related to our RRR question.

## 5. Conclusions

This rapid review was conducted alongside a scoping review to explore the contextual factors and mechanisms associated with mental health interventions for individuals with chronic physical health conditions at risk from COVID and severe illness. A socio-ecological framework with macro-meso-micro systems levels served as the programme theory. This review suggests that trust, accountability, social connectedness, resilience and power-sharing are key mechanisms associated with policy action (macro level), primary care provider and community-based service provision (meso level), and individual and family uptake of services (micro level). This review provides testable CMO explanations at each systems level that may be associated with relevant, sustainable mental health interventions for vulnerable populations. 

## Figures and Tables

**Table 1:** Fourteen scoping review documents used in the rapid realist review.

Authors and Year	Country	Topic of Paper
Chevance et al. 2020 [10]	France	Ensuring mental health care
Chakraborty 2020 [17]	UK	Mental health exacerbation
Khan et al. 2020 [18]	China	Psychological health
Wang et al. 2020 [19]	China	Blood glucose management
Razai et al. 2020 [20]	UK	Mitigating social isolation
Pulvirenti et al. 2020 [21]	Italy	Remote assistance for immunodeficient patients
Casale and Flett 2020 [22]	Italy, Canada	Interpersonally based fears
Goodman-Casanova et al. 2020 [23]	Spain	Telehealth home support
Padala et al. 2020 [24]	US	FaceTime use with Alzheimer’s patients
Rozanova et al. 2020 [25]	Ukraine	Social support for vulnerable seniors with HIV and substance use
Rodler et al. 2020 [26]	Germany	Telehealth
Umucu and Lee 2020 [27]	US	Coping strategies
Vanni et al. 2020 [28]	Italy	Decision-making process
Chong et al. 2020 [29]	Australia	Social isolation and older adults

**Table 2:** Mechanisms and context–mechanism–outcomes at macro–meso–micro systems levels during COVID-19.

Mechanism	Socioecological Level	Context–Mechanism–Outcome (CMO)
Trust	Macro Policy/Government	When the government ensures timely access to valid information and mental health support services (C), negative emotions, such as anxiety and fear are decreased (O) due to trust (M) in the government’s capacity to meet public needs.
	Meso Community/Primary Care	When well-known, established community services and providers are used to promote mental health interventions (C), public engagement and uptake of services is increased (O), due to community/provider trust (M).
	Micro Individual/Family	When support workers already have relationships with clients, their families and carers (C), clients and families are more apt to follow guidance (O) due to trust (M) in worker knowledge of their specific needs.
Social Connectedness	Macro Policy/Government	When the government funds volunteer and trained staff outreach (C), at-risk individuals (e.g., isolated seniors in their homes) are at decreased risk for mental and physical health deterioration (O) due to social connectedness (M).
	Meso Community/Primary Care	When primary care providers use social prescribing with patients (C) patients are better able to meet their mental and physical health needs (O), because they are socially connected (M) to a range of community services.
	Micro Individual/Family	When individuals are at risk of emotional and behavioural difficulties due to isolation (C), negative experiences from confinement are reduced (O) by staying socially connected (M) via support networks and technology use.
Accountability	Macro Policy/Government	When public health officers provide factual, timely information to the media (C) the public concerns about COVID-19 are decreased (O) due to government accountability for communications about public health response.
Resilience	Macro Policy/Government	When regulatory and communications barriers are lowered (C), the public has means to stay social connected (O) due to government resilience (M)
	Meso Community/Primary care	When primary care providers and community services are re-organised to consider access of services for at-risk patients (C), recurrences of mental health exacerbations can be decreased (O) due to service resilience (M). When primary care providers use mental health screening tools with patients (C), proactive mental health promotion strategies can be implemented (O) due to resilient attention (M) to increased anxiety and depression during COVID-19
	Micro Individual/Family	When individuals learn how to use adaptive coping strategies, such as acceptance and self-distraction (C), there are lower levels or negative emotions from COVID-19 (e.g., anxiety, depression (O) due to personal resilience (M).

**Table 3:** Eleven (pre-COVID) realist reviews identified in supplementary searches.

Authors and Year	Country	Topic Focus of Review
Abayneh et al. 2018 [30]	Ethiopia	Service user and caregiver involvement
Blair et al. 2014 [31]	Canada	Neighbourhood variables and depression
Dalkin et al. 2018 [32]	UK	Impact of intensive advice services on health
De Weger et al. 2018 [33]	The Netherlands	Community engagement in developing health and care systems
Gray et al. 2019] [34]	South Africa, and Canada	Promoting mental health and wellbeing among healthcare workers
Husk et al. 2020 [35]	UK	Approaches to social prescribing
James et al. 2020 [36]	Indonesia/UK	Civic engagement within mental health services
Keady et al. 2012 [37]	UK	Neighbourhood variables and dementia
Lamontagne-Godwin et al. 2018 [38]	UK	Physical health screening in people with severe mental illness
O’Campo et al. 2009 [39]	Canada	Community-based services for homeless adults experiencing concurrent mental health and substance use disorders
Tyler et al. 2019 [40]	Canada	Social paediatric initiatives

**Table 4:** Policy and practice recommendations for mental health interventions.

Mechanism	Summary	Recommendation
Trust	Trust depends on pre-existing relationships or networks. Trust relationships across levels depend on timely access to needed information and services.	Policy makers and practitioners implementing mental health interventions are more likely to develop effective programs if they, first and foremost, invest in ongoing and long-standing relationships with key stakeholders with whom they share decision-making. This will allow the programs to be more appropriate, responsive, resilient to crisis, and to have greater uptake.
Accountability	Stakeholder engagement increases accountability and uptake of collaboratively planned services.	Accountability at each systems level can be promoted by engaging key stakeholders in shared decision-making. However, it is important to consider using models of community engagement and participatory models which aim to ‘level’ the playing field between stakeholders.
Social Connectedness	Social connection is vital to improved health and well-being.	Technological innovations and services, such as social prescribing need to be formalised, advertised, and promoted at each systems level.
Resilience	The pandemic triggered quick, responsive organisational and service resilience. Sustainable resilience may depend on relational reserves and long-standing, and ongoing relationship-building with key stakeholders, especially users.	Building and maintaining resilience should be a focus across all levels of complex health systems, with on-going examination and mitigation of stresses and upstream/downstream impacts.
Power-sharing	Although we did not find this mechanism in our RRR of the scoping review literature during the pandemic, we believe that this mechanism is closely related to the other mechanisms, especially trust, accountability, and resilience.	The best way to share power at each systems level is via participatory models of planning, implementation and evaluation of mental health services.

## Data Availability

Not applicable.
